# Patients’ Perspective on Termination of Pacemaker Therapy—A Cross-Sectional Anonymous Survey among Patients Carrying a Pacemaker in Germany

**DOI:** 10.3390/healthcare11212896

**Published:** 2023-11-03

**Authors:** Irene Portig, Elif Karaaslan, Elena Hofacker, Christian Volberg, Carola Seifart

**Affiliations:** Research Group Medical Ethics, Faculty of Medicine, Philipps University of Marburg, 35043 Marburg, Germany; portig@uni-marburg.de (I.P.); hofacker@uni-marburg.de (E.H.); zwiebel@uni-marburg.de (C.S.)

**Keywords:** pacemaker, end-of-life care, patients’ perspective, autonomy

## Abstract

Objective: To determine the opinions of patients regarding the withdrawal of pacemaker therapy. Participants and methods: A cross-sectional anonymous questionnaire was administered to patients visiting an outpatient cardiologic clinic for routine follow-up visits of pacemaker therapy or patients carrying a pacemaker admitted to a hospital between 2021 and 2022. Results: Three-hundred and forty patients answered the questionnaire. A total of 56% of the participants were male. The mean age was 81 years. The majority of respondents were very comfortable with their PM and felt well informed, with one exception: more than half of respondents were missing information on withdrawal of pacemaker therapy. Almost two-thirds wanted to decide for themselves if their pacemaker therapy was withdrawn regardless of whether they were ill or healthy. Almost 60% of patients would like the pacemaker to be turned off when dying. Women expressed this wish significantly more often than men. Conclusion: Our survey shows that patients prefer to be informed on issues regarding the withdrawal of pacemakers as early as preimplantation. Also, patients would like to be involved in decisions that have to be made at the end of life, including decisions on withdrawal. Offers of conversations about this important issue should include information on special features of the patient’s pacemaker, e.g., the absence or presence of pacemaker dependency. Knowledge about the pacemaker’s functionality may prevent distress among individuals nearing their end of life when, for example, under the false impression that timely deactivation may allow for a more peaceful death.

## 1. Introduction

The first pacemaker (PM) was implanted in 1958. This technology was continuously developed and later supplemented by an implantable cardioverter deactivator (ICD) and cardiac resynchronization therapy (CRT). In Germany, the number of newly implanted cardiac implantable electronic devices (CIED) increased continuously over the years and has been decreasing slightly since 2016 [1,2]. This is explained by more cautious indications and improvements in the prevention and treatment of heart failure. Currently, the number of new PM implantations in Germany is relatively stable at around 75,000 per year (approximately 900 per million inhabitants), with around two thirds of patients aged 70 years or older, though this is expected to grow in an aging population.

Pacemaker therapy is indicated in the presence of bradyarrhythmia, where patients with a high degree of atrioventricular block (AVB) have notably poorer survival compared to patients treated with a pacemaker [2,3]. In sinus node dysfunction, on the other hand, the second most common indication, pacemaker therapy, does not seem to improve prognosis [4]. More importantly, PM therapy has been shown to improve the quality of life of treated patients considerably.

Mortality amongst recipients of PM has been reported to amount to 20% per year [5], who are likely to die either from the progression of their cardiac disease or, in up to 50% of cases, from non-cardiac terminal illness. Therefore, healthcare professionals, including those without cardiologic expertise, will, in the future, have to treat patients asking for the withdrawal of their PM and should be aware of the issues associated with device deactivation, including ethical and legal implications.

The US American Heart Rhythm Association issued expert consensus statements affirming the ethical and legal permissibility of CIED deactivation, even if the patient was not terminally ill [6]. This statement was developed in collaboration with members of the European Heart Rhythm Association. The equivalent European consensus statement focuses on the deactivation of implantable cardioverter-defibrillators (ICD) in dying patients [7]. It states that less agreement exists in Europe for PM deactivation. In Germany, an equivalent statement was published in 2017 regarding the withdrawal of (ICD) but not pacemaker therapy [8]. However, it should be pointed out that there are various approaches in the literature to define the term “terminally ill”, and a commonly accepted definition does not exist [9,10,11]. In Germany, it refers to the last phase of life in the context of a chronic, life-threatening illness, which can extend over a period of weeks to a few months.

Most healthcare professionals regard device deactivation in dying patients as allowing natural death, especially when intended to alleviate symptoms and not hasten death [12,13,14,15,16,17,18]. However, in PM-dependent patients, defined as the absence of an intrinsic rhythm during the inhibition of pacing [19], a number of physicians object to deactivating PMs, arguing that it can either lead to symptoms of heart failure or death [6,16,20].

Over the past decade, concerns have been expressed about non-beneficial or medically futile treatment in middle and high-income countries [9,21,22,23]. Also, the appropriateness of existing treatment strategies has become a matter of debate and includes decisions on the discontinuation of such treatment at the end of life. In our view, these discussions should include pacemaker therapy, for example, by working out criteria for termination, even in PM dependency.

Since end-of-life care for patients can be challenging, and little is known about patients’ opinions and attitudes toward the withdrawal of pacemaker therapy, a cross-sectional survey was conducted in Germany among patients carrying a pacemaker to fill this gap.

## 2. Materials and Methods

Between May 2021 and July 2022, a cross-sectional anonymous questionnaire was administered to patients carrying pacemakers (PM). Our cross-sectional survey was conducted using a questionnaire with closed-ended questions to capture participants’ experience with their PM.

The items were developed using a two-step methodology. First, a multidisciplinary research team drafted items that were based on scientific knowledge, a survey of the literature, and their own experiences from clinical practice through an iterative consensus procedure. During these discussions, the research team agreed on items inquiring about the general perception of their PM and secondly on items regarding experiences with, attitudes toward, and knowledge of issues in relation to the implantation of, life with, and decisions about the deactivation of PM.

Second, prior to the final application of this questionnaire, cognitive interviews were undertaken with three patients that helped to test problems on feasibility, e.g., the comprehensibility of questions or acceptance. Minor changes in wording were made to the questionnaire according to the results obtained.

### 2.1. Data Collection

During the main study, the final questionnaire was read aloud to each participant. The interviewer filled in the questionnaire manually according to the answers given. The following socio-demographic variables were collected: patient’s age, sex, and year of implantation.

### 2.2. Study Population and Recruitment

Our study targeted patients appearing for the follow-up of their pacemaker at the outpatient device clinic at the local university hospital and a local cardiologist’s practice characterized by an urban as well as a rural environment. In addition, patients with PM who were admitted to the local university hospital, and three teaching hospitals were included. The following eligibility criteria applied to our study:

Inclusion criteria were adult patients carrying a pacemaker, sufficient German language comprehension, willingness to participate, and an ability to give consent.

Exclusion criteria were hearing or visual impairment of severity that interfered with interviewing.

The study was conducted during the COVID-19 pandemic, and recruitment had to be interrupted several times due to restrictions on conducting research projects.

The study was approved by the local Ethics Committee for Human Research at Philipps University Marburg, Marburg, Germany (161-20), and was registered with the German Registry for Clinical Studies (DRKS00026168). The trial was conducted in accordance with the principles of the Declaration of Helsinki.

### 2.3. Statistical Analysis

Statistical analysis was carried out descriptively using SPSS (version 29, 2022) and group comparisons by means of a chi-square test according to Pearson. Possible influencing factors were calculated using univariate and multivariate logistic regression models.

## 3. Results

A total of 340 patients were included during the restricted recruitment periods. In total, 56% of the participants were male. The mean age was 81 years (SD 11.4). The youngest participant was 20, and the oldest 99 years old.

### 3.1. General Perception of PM

**Figure 1:**
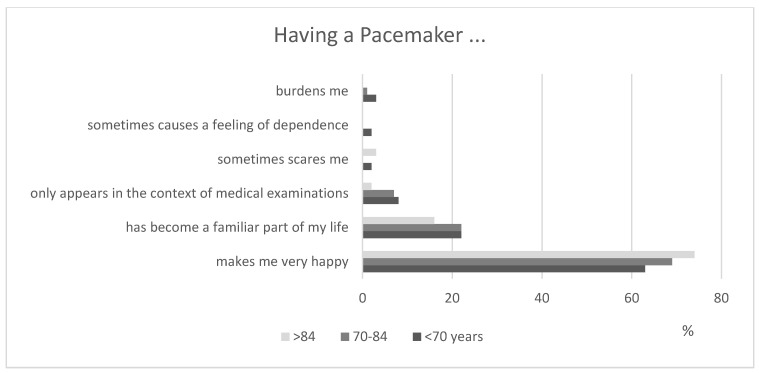
Patients’ attitudes toward PM therapy.

An evaluation of 340 questionnaires confirmed that patients were, by and large, very pleased with their pacemaker (*n* = 305/92%). Most of them perceived it “as a great luck, because it helps to live a good life”. This positive feeling increased with age (Figure 1).

Patients rarely (*n* = 55 (16%)) or never (*n* = 217 (64%)) thought about their PM in everyday life. Only 28 (7%) stated that the PM often occupies their thoughts. Sixteen (4.7%) participants reported problems with their PM, such as local pain, some discomfort through the wearing of bras, or movement restrictions.

### 3.2. Issues Regarding Withdrawal of PM

**Figure 2:**
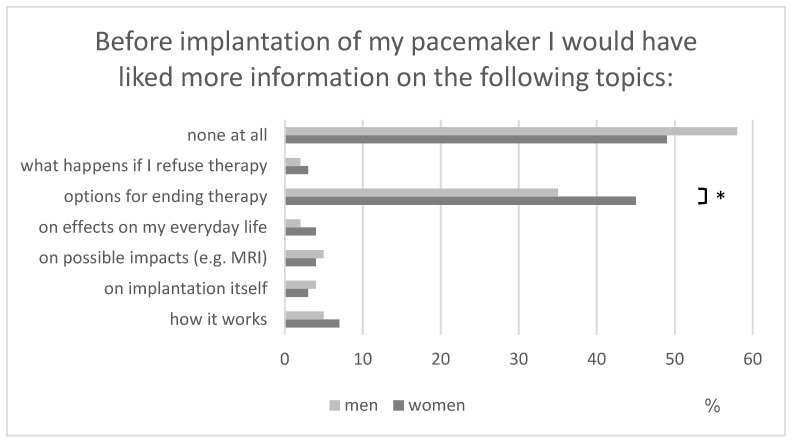
Patients’ desire for more information regarding their PM (* *p* < 0.05).

Before implantation, the vast majority felt well informed (*n* = 237/70.5%), especially regarding aftercare (*n* = 306/91%) and particularities of living with a PM (*n* = 226/67%). This impression was confirmed when asked, which topics should be explained in more detail before implantation with one exception: 134 (40%) answered that they missed information about withdrawal therapy. This issue was more important for women than men (Figure 2) and for older than younger individuals. It was irrelevant whether respondents were outpatients or admitted to hospital.

At the time of implantation, 27 (8%) patients and, thereafter, 42 (14%) patients wondered if device therapy could be withdrawn upon their request. In total, 34 (11%) patients remembered that they had been informed about the possibility of deactivation. Fifty-eight (17%) patients were worried that the PM might keep them alive longer than necessary (Table 1). This latter concern was more frequently expressed by outpatients than patients admitted to the hospital.

**Figure 3:**
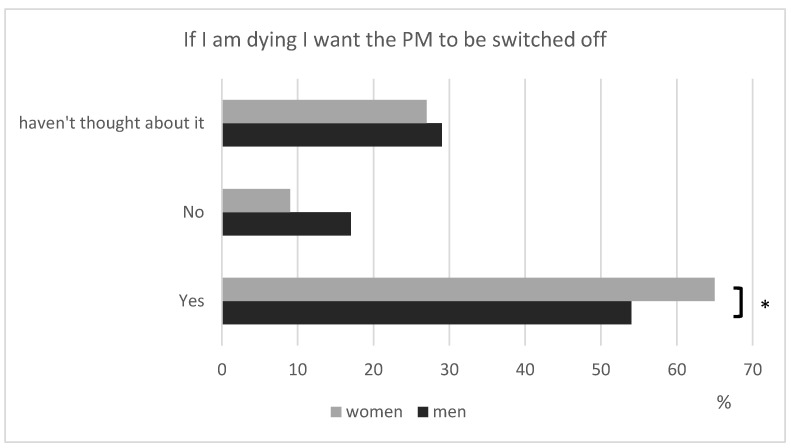
Patients’ desire for PM withdrawal at the end of life (* *p* < 0.05).

In total, 199 (59%) patients would like the PM to be turned off when dying, a concern voiced by women more often than men, regardless of age (Figure 3).

**Figure 4:**
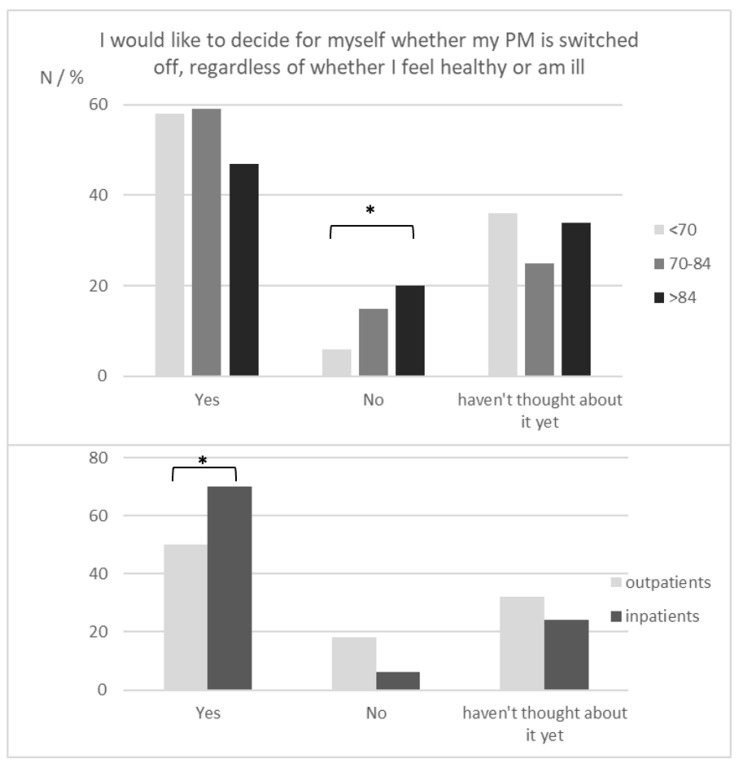
Patients’ desire for an autonomous decision regarding PM withdrawal (* *p* < 0.05).

A total of 190 (56%) patients wanted to decide for themselves whether their PM was deactivated, regardless of whether they were healthy or ill. Patients admitted to the hospital expressed this wish more often than outpatients in younger people less often than older people (Figure 4).

## 4. Discussion

Our study provides important insights into patients’ wishes regarding information given by health care professionals concerning their pacemaker (PM) therapy, including withdrawal at the time of implantation and during follow-up visits and their involvement in end-of-life discussions, including the possibility of the withdrawal of PM therapy. We would like to point out at this point that we cannot provide any information on the question of what happens if a person is no longer capable of giving consent at the end of life. This topic is very comprehensive, differs in various countries due to the prevailing legal situation, and cannot be answered with available data from our survey.

### 4.1. Information on Withdrawal of Therapy as Part of the Shared Decision-Making Process

In contrast to ICDs or CRT-Ds, where it is recommended to explain the possibility of deactivation before implantation if applicable, this kind of discussion is—according to published consensus statements—not necessary in PM patients or even as part of end-of-life (EOL) care, unless requested by the patient [24].

In our study, a high percentage of patients declared that they had not received but would have liked more information about the possibility of deactivation before their PM was inserted. This finding is in accordance with previous work where even patients with ICDs had limited knowledge about withdrawal [12,13,25,26,27,28].

Informed consent requires that patients understand the risks, benefits, and consequences of interventions in light of what is important to them in making such decisions [29]. Following the results of the present study, this should include information on withdrawal from therapy even if deemed unnecessary or unreasonable by health care professionals. Patients and those close to them often have misconceptions about what will happen if they receive a PM or if their PM is turned off. Possible scenarios and reasons for the healthcare professionals’ assessments should be communicated and include, for example, uncertainty as to the immediate effects of device deactivation in PM-dependent patients. If these conversations occur as early as around the time of implantation, unjustified fears as to unnecessary suffering (e.g., prolonging the process of dying) from PM or unrealistic hopes (e.g., prolonging life even with poor health status) may be overcome or may not arise at all.

### 4.2. Withdrawal of PM Therapy

More than half of the participants in our study would like to decide for themselves if the PM is withdrawn, regardless of whether they are healthy or ill. In our study, a standardized questionnaire was used, which did not allow for the exploration of respondents’ motivation. We believe that this might be due to the current discourse on assisted suicide in Germany, giving this topic more attention and thereby probably leading to more confidence in self-determined decisions. This wish for autonomous decision-making was more prevalent in the group of patients where the mean age was 81 years, but again underlines the importance of a shared decision-making process where patients’ wishes and preferences are heard and respected. In line with this finding, patients admitted to hospitals and older persons, where EOL discussions are more likely to occur, express this wish more often.

A meta-systematic review tried to identify common themes able to act “as the foundation for efforts … in improving the way we die” [30]. They found that the following themes were deemed necessary for a “good death” in Western society: autonomy with regard to treatment-related decision making; life not being prolonged unnecessarily; and the right to terminate one’s life. We believe that our results affirm these findings since a large proportion of participants in our survey made an informed and autonomous decision about their PM therapy.

Additionally, in the said review, effective communication with professionals and an awareness of the deep significance of what is happening were named. This again highlights the necessity for healthcare professionals to ensure that patients understand the reasons behind their recommendations but also that medical professionals are able to address patients’ fears and concerns and understand the decisions for or against a proposed therapy.

When discussing the termination of PM therapy, both the positive and the negative aspects of the process must be mentioned by the physician. In pacemaker-dependent patients, deactivation may lead to immediate death, which is very likely to be the intention of the person with terminal disease. However, it can also happen that a junctional rhythm develops, and the patient might suffer additionally through dyspnea or severe dizziness due to bradycardia. These and other aspects should be discussed in detail with the patient and his or her relatives, and the deactivation should be carried out in a protected setting, preferably with the support of a palliative care physician.

### 4.3. Withdrawal of PM Therapy at the End of Life (EOL)

Almost two-thirds of the respondents in our study want their PM to be turned off when dying. It is speculated that the respondents’ wish for withdrawal is based on the desire for a self-determined decision. But this may also result from misunderstandings on the possible impact of the PM on the duration of or suffering during the dying process. In this context, many people believe that the dying process is prolonged by a PM, although this is not the case. At this point, a distinction must be made between PM and ICD therapy. With an ICD, suffering can be prolonged if electric shocks are delivered during the dying process and if the heart rhythm turns into ventricular tachycardia or ventricular fibrillation. A pacemaker, however, cannot further stimulate a lifeless heart and consequently has no effect on increased suffering.

Interestingly, women opted for withdrawal significantly more often than men. In accordance with our findings, various studies reported that women have a greater likelihood of “do not attempt resuscitation” (DNAR) use in acute critical illnesses, such as cardiac arrest or intracerebral hemorrhage [31,32,33]. A DNAR order limits aggressive life-restoring treatment such as cardiopulmonary resuscitation and is not an order to limit treatment. Although the reasons for this finding are unknown, it has been proposed that women have talked more often than men to their loved ones about their wishes should a crisis occur.

It has also been reported that men are more reluctant than women to enter into discussion regarding their own impending death [34], e.g., via advance care planning [35,36]. This gender difference has been ascribed to behavior patterns traditionally attributed to men, such as self-reliance and restrained emotionality [37]. Most interestingly, Skulason found that men were, in principle, willing to discuss EOL issues, but these had to be coaxed out in conversation [38]. The authors conclude that “gender differences in terminal care communication may be radically reduced by using simple relatively unpretentious evocation methods”, but these “require considerable clinical training” [38].

## 5. Limitations

Apart from the known general limitations of cross-sectional surveys, such as deductive assumptions in the conception of the questions, this study has several additional limitations. The group under study is not representative of PM patients in general. Patients were recruited in an outpatient clinic of a tertiary referral center and in a university hospital and its teaching hospitals in a rural area in Germany. Moreover, patients were recruited during the COVID pandemic, the survey had to be interrupted several times, and a number of patients canceled their appointments during the pandemic to avoid unnecessary contact. Our results cannot, therefore, be transferred to other clinical settings.

## 6. Conclusions

Our survey shows that patients prefer to be informed on issues regarding withdrawal from PM as early as preimplantation. Also, patients would like to be involved in decisions that have to be made at their end of life, including decisions on the withdrawal of a PM, even if not nearing EOL.

Opportunities to hold conversations about this important issue are clearly warranted, including information on the special features of the patients’ PM, e.g., the absence or presence of PM dependency, which should probably start as early as around the time of implantation and be regularly updated at follow-up visits. Additional studies are required to examine how best to approach the observed gender and age effect.

Knowledge about the functionality of PMs may prevent distress among persons nearing EOL and their loved ones (and health care professionals) when under the false impression that timely PM deactivation allows for a more peaceful death.

## Figures and Tables

**Table 1:** Patients’ concerns regarding and knowledge about withdrawal from PM therapy.

Withdrawal of PM	*N*	Yes	No	Undecided
***BEFORE IMPLANTATION***				
Information received	338	37 (11%)	287 (85%)	14 (4%)
Wish for more information	333	134 (40%)	199 (60%)	0 (0%)
Personal thoughts about withdrawal	338	27 (8%)	295 (87%)	16 (5%)
***SINCE IMPLANTATION***				
Thoughts about possibility of	338	42 (12.4%)	290 (85.8%)	6 (1.8%)
Wish for withdrawal at end of life	338	199 (59%)	44 (13%)	95 (28%)
Wish for autonomous decision, regardless of terminal illness	339	190 (56%)	49 (14.5%)	100 (29.5%)
Concerns about PM preventing death	336	58 (17%)	279 (83%)	0 (%)
		Often	Rarely	Never/Undecided
Frequency of these concerns	338	9 (3%)	84 (25%)	245 (72%)

## Data Availability

Data are available on request. A request can be made to the corresponding author.
